# Demand and use of health services by Brazilian adolescents, according to the National School Health Survey 2019

**DOI:** 10.1590/1980-549720230008.supl.1

**Published:** 2023-04-21

**Authors:** Alanna Gomes da Silva, Crizian Saar Gomes, Alan Cristian Marinho Ferreira, Deborah Carvalho Malta

**Affiliations:** I Universidade Federal de Minas Gerais, Nursing School, Graduate Program in Nursing – Belo Horizonte (MG), Brazil.; II Universidade Federal de Minas Gerais, Medical School, Graduate Program in Public Health – Belo Horizonte (MG), Brazil.

**Keywords:** Adolescent health, Health services accessibility, Unified health system, Health promotion

## Abstract

**Objective:**

To analyze the demand and use of health services by Brazilian adolescents, according to sociodemographic characteristics.

**Methods:**

Cross-sectional study with data from the 2019 National School Health Survey, that assessed 124,898 adolescents aged 13 to 17 years. The crude and adjusted prevalence ratios (RPaj) by sex, age, and school administrative status and their 95% confidence intervals (95%CI) were calculated for the variables “search for a service or health professional”, “search for a Basic Health Unit” and “assistance at the Basic Health Unit”, using Poisson regression with robust variance.

**Results:**

The demand for a health service was reported by 56.56% (95%CI 55.82–57.29) of the adolescents and was lower among male students (RPaj: 0.95; 95%CI 0.94–0.95); those with black skin color (RPaj: 0.95; 95%CI 0.94–0.97), brown skin color (RPaj: 0.97; 95%CI 0.96–0.98), yellow skin color and indigenous ethnicity (RPaj: 0.95; 95%CI 0.94–0.97); public school students (RPaj: 0.90; 95%CI 0.89–0.90); and rural residents (RPaj: 0.96; 95%CI 0.94–0.98). A Basic Health Unit was the service sought by 74.08% (95%CI 73.21–74.94) of adolescents, more frequently among students of brown skin color (RPaj: 1.06; 95%CI 1.03–1.08), from public schools (RPaj: 1.32; 95%CI 1.29–1.35) and residing in rural areas (RPaj: 1.05; 95%CI 1,01–1,09). The main reason for seeking the Basic Health Unit was vaccination (27,93%; 95%CI 27,07–28,81).

**Conclusion:**

More than half of the adolescents searched for a health service, which means that this group has a high demand. However, health inequalities still persist and point to the importance of health care planning, reception conditions, and the quality of care provided.

## INTRODUCTION

Individuals go through a dynamic and complex process of maturation and physical, hormonal, psychic and social transformations during adolescence, which can predispose them to new experiences and health-risky behaviors^1,2^.

Every year, thousands of adolescents die from preventable causes^3^, which reinforces the need for a comprehensive health care and access to individual and collective health promotion actions, as well as disease and injuries prevention programs^4,5^. In order for adolescents to be fully cared for, an organized, planned network with the participation of social actors and health professionals trained to assist them is needed; one that considers, above all, socioeconomic and cultural contexts^6^.

The search for a health service can be defined as the entry of an individual into the health system, while use comprises the interaction between professionals and users, be it direct contact, such as consultations and hospitalizations, or indirect contact, such as performing exams^7^.

The demand and use of health services can be influenced by social determinants, once low-income, socially disadvantaged or marginalized adolescents are less likely to seek health services. On the other hand, white adolescents, enrolled in private schools and with high maternal educational level seek health services and professionals more often^8,9^. This is added to the difficulty of professionals in providing comprehensive care to adolescents, which impairs reception, bonding and the quality of care offered^4^.

Studies using data from the National School Health Survey (PeNSE) showed that 48.0% (95%CI 47.6–48.5) of adolescents looked for a health service or professional in 2012^8^ and 56.7% (95%CI 55.2–58.3) in 2015^9^, which shows an increase over the years. However, the monitoring of demand and use of health services must be continuous to enable decision-making, improve management and public policies and recognize the demands of this population, while identifying factors that influence the relationship with services and health professionals. Thus, it is expected that continuous, coordinated and guided care to adolescents will be promoted and improved, ensuring their rights and reducing health inequalities.

Therefore, this study aimed to analyze the demand for and use of health services by Brazilian adolescents, according to sociodemographic characteristics.

## METHODS

### Study design

This is a cross-sectional study that used data from the PeNSE 2019 survey.

### Scenario

PeNSE is a survey carried out with adolescents in school age, conducted by the Brazilian Institute of Geography and Statistics (IBGE) in partnership with the Ministry of Health and supported by the Ministry of Education. It is part of the Surveillance System for Risk and Protection Factors for Chronic Noncommunicable Diseases (NCDs) in Brazil and was the first national survey to address various aspects of adolescents’ lives, such as habits, care, risk and protection factors for health^10^.

The sampling plan was by conglomerates in two stages, in which schools corresponded to the first stage of selection and the groups of students enrolled corresponded to the second stage. The set of students from selected classes formed the sample. The plan was designed to estimate population parameters for students aged 13 to 17 years who were enrolled and regularly attending public and private schools, for the following geographic levels: Brazil, major regions, Federation Units (FU), capital cities and the Federal District. Details on the sampling process are provided in the PeNSE official publication^10^.

In 2019, data were collected at 4,242 schools, 6,612 classes, from 189,857 students enrolled and 183,264 attending students, 159,245 valid questionnaires and 125,123 questionnaires analyzed^10^.

PeNSE's database and questionnaires are available in public domain and for use on the IBGE website: https://www.ibge.gov.br/estatisticas/sociais/saude/9134-pesquisa-nacional-de-saude-do-escolar.html?edicao=31442&t=results.

### Participants

Adolescents aged 13 to 17 years old, enrolled and regularly attending the 7^th^ to 9^th^ grade of elementary school and the 1^st^ to 3^rd^ grades of high school, including technical courses with integrated high school and normal/teaching courses, participated in the research; participants were from all shifts, from public and private schools in Brazil^10^.

### Data collection

Data were collected from April to September 2019 by means of the mobile collection device, which corresponds to a smartphone with the structured survey questionnaires. The IBGE technician distributed the devices to students present on the day of the interviews and instructed them on how to handle them. The student's questionnaire was self-administered and had specific fill-in instructions^10^.

### Study variables

This study included variables related to demand and use of health services and sociodemographic features that are described below.

Search for a health service or professional: prevalence of adolescents who sought a health service or professional to care for their health. “In the last 12 months, have you sought any health service or professional to care for your own health?” Answer options: yes; no.

Most frequently sought service: proportion of type of health service sought most frequently by adolescents to care for their health. “In the last 12 months, which health service did you visit most often?” Response options: Basic Health Unit (BHU); doctor's office or private clinic; hospital; others (dental office; office of other health specialty; medical specialty services or polyclinic; emergency room, Emergency Care Unit; laboratory or clinic for complementary tests; home care service; pharmacy).

Search for a BHU: proportion of adolescents who sought a BHU to care for their health in the last 12 months. “In the last 12 months, have you sought any BHU (Health Center or Family Health Unit/Family Health Strategy — FHS)?” Answer options: yes; no.

Assistance at the BHU: proportion of adolescents who were assisted when they sought a BHU. “Did you receive care the last time you went to a BHU (Health Center or Family Health Unit — FHS)?” Answer options: yes; no.

Reason for looking for a BHU: proportion of main reason adolescents sought a BHU. “What was the main reason for your visit to the BHU (Health Center or Family Health Unit — FHS) this last time?” Answer options: weight management support (gain or lose); accident or injury, rehabilitation or physical therapy; dentist or other oral health professional; psychologist or other mental health professional; vaccination; illness; others (support to smoking cessation; contraceptive methods; HIV, syphilis or hepatitis B test; prenatal care/pregnancy test; request for a medical certificate).

**Figure 1:**
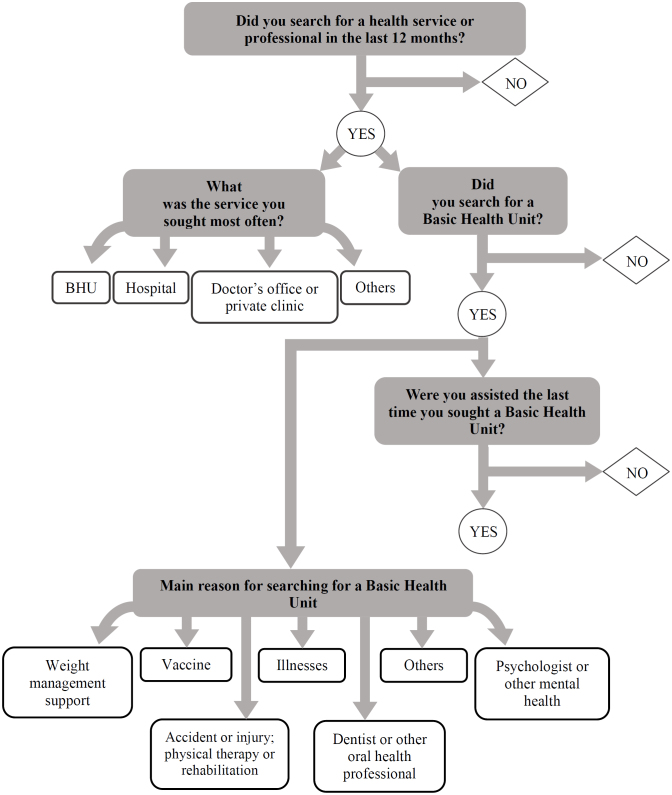
Flowchart of questions related to the search for and use of health services by students aged 13 to 17 years. PeNSE, Brazil, 2019.

Figure 1 shows the flow of the PeNSE questionnaire regarding the aforementioned indicators.

The sociodemographic variables were: biological sex (male and female); age range (from 13 to 15; 16 and 17 years old); skin color (white, black, brown and others — yellow and indigenous); school network (private and public), and area of residence (urban and rural).

### Data analysis

Prevalence values and respective 95% confidence intervals (95%CI) were estimated. For the variables “search for a health service or professional”, “search for a BHU” and “assistance at the BHU”, proportions were calculated according to sociodemographic variables and crude and adjusted prevalence ratios (PRc and PRa) by sex, age and school network^8,9^ and respective 95%CI, using Poisson regression models with robust variance. Results with p-value lower than or equal to 0.05 were considered significant. For all analyses, post-stratification weights were considered.

Analyses were performed using Data Analysis and Statistical Software (Stata), version 14.2, by means of the survey module, which considers post-stratification weights.

### Ethical aspects

The students who agreed with the Free and Informed Consent Form, displayed on the first page of the questionnaire in the mobile collection device, participated in the research.

PeNSE was approved by the National Committee on Ethics in Research for Human Beings of the Ministry of Health (Opinion No. 3,249,268, of April 8, 2019).

## RESULTS

A total of 124,898 adolescents aged between 13 and 17 years were evaluated. Of these, most were females (50.7%; 95%CI 49,9–51.4); aged between 13 and 15 years (64.7%; 95%CI 63.2–66.01); with brown skin color (43.6%; 95%CI 42.8–44.3), followed by white (36.0%; 95%CI 35.1–36.8), black (13.6%; IC95% 13,0–14.1), yellow skin and indigenous ethnicity (6.9%; 95%CI 6.5–7.2); living in the urban area (92.4%; 95%CI 90.6–93.6); and enrolled in public schools (85.5%; 95%CI 85.1–85.9).

The search for a health service or professional to care for their health was reported by 56.56% (95%CI 55.82– 57.29) of the adolescents and was lower among males (PRa: 0.95; 95%CI 0.94–0.95); those whose skin was black (PRa: 0.95; 95%CI 0.94–0.97), brown (PRa: 0.97; 95%CI 0.96–0.98), yellow, and those who were indigenous (PRa: 0.95; 95%CI 0.94–0.97); those enrolled in public schools (PRa: 0.90; 95%CI 0.89–0.90); and living in rural areas (PRa: 0.96; 95%CI 0.94–0.98). On the other hand, demand was higher among adolescents aged 16 and 17 years (PRa: 1.03; 95%CI 1.02–1.04) (Table 1).

**Table 1:** Search for a health service or professional to care for their own health, according to sex, age, skin color, school status and place of residence. National School Health Survey, Brazil, 2019.

		Search for a health service or professional		PRc (95%CI)	PRa (95%CI)
		Yes % (95%CI)	No % (95%CI)		
Total		56.56 (55.82– 57.29)	43.44 (42.71–44.18)		
Sex					
	Female	60.72 (59.85–61.58)	39.28 (38.42–40.15)	*	*
	Male	52.23 (51.27–53.19)	47.77 (46.81–48.73)	0.95 (0.94–0.95)	0.95 (0.94–0.95)
Age (years)					
	13–15	55.12 (54.08–56.14)	44.88 (43.86–45.92)	*	*
	16–17	59.20 (58.28–60.12)	40.8 (39.88–41.72)	1.03 (1.02–1.04)	1.03 (1.02–1.04)
Skin color					
	White	61.25 (60.16–62.34)	38.75(37.66–39.84)	*	*
	Black	51.45 (49.64–53.25)	48.55 (46.75–50.36)	0.94 (0.93–0.95)	0.95 (0.94–0.97)
	Brown	55.03 (54.03–56.02)	44.97 (43.98–45.97)	0.96 (0.95–0.97)	0.97 (0.96–0.98)
	Others	51.93 (49.57–54.27)	48.07 (45.73–50.43)	0.94 (0.93–0.96)	0.95 (0.94–0.97)
Status					
	Private	71.55(70.73–72.36)	28.45 (27.64–29.27)	*	*
	Public	54.02 (53.16.54.87)	45.98 (45.13–46.84)	0.90 (0.89–0.90)	0.90 (0.89–0.90)
Residence area					
	Urban	57.27 (56.50–58.04)	42.73 (41.96–43.5)	*	*
	Rural	47.9 (45.13–50.68)	52.1 (49.32–54.87)	0.94 (0.92–0.96)	0.96 (0.94–0.98)

* Reference group for calculating the prevalence ratio. %: prevalence; 95%CI: 95% confidence interval; PRc: crude prevalence ratio; PRa: prevalence ratio adjusted for sex, age and school status

**Figure 2:**
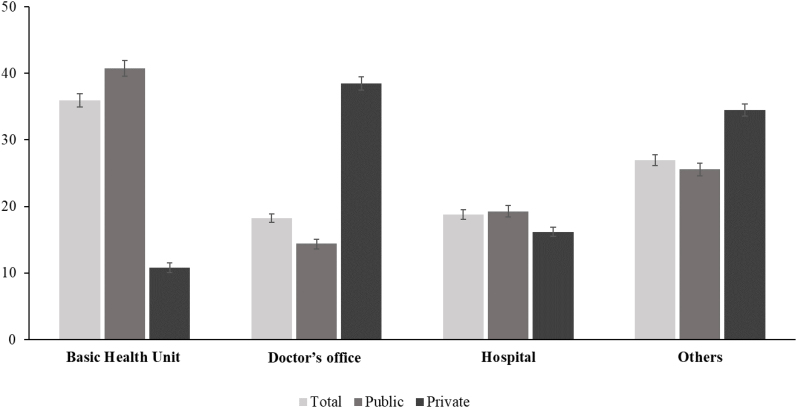
Type of service sought most frequently by students aged 13 to 17 years for care related to their own health, according to administrative status. National School Health Survey, Brazil, 2019.

Regarding the type of health service most frequently sought by adolescents, the results indicated that 35.97% (95%CI 34.98–36.97) looked for the BHU. It is noteworthy that public school students sought the BHU more frequently (40.75%; 95%CI 39.56–41,95) and those from private schools sought a doctor's office or private clinic (38.49%; 95%CI 37.52–39.46) (Figure 2).

When asked specifically about searching for a BHU, 74.08% (95%CI 73.21-74.94) of the adolescents sought care at this service, mainly brown-skinned students (PRa: 1,06; IC95% 1,03–1,08), from public schools (PRa: 1.32; 95%CI 1.29–1.35) and residing in rural areas (PRa: 1.05; 95%CI 1.01–1.09). By contrast, the BHU was less sought by males (PRa: 0.94; 95%CI 0.92–0.96). We found no difference according to age group (Table 2).

**Table 2:** Schoolchildren aged 13–17 years old who searched for a Basic Health Unit, according to sex, age, skin color, school status and area of residence. National School Health Survey, Brazil, 2019.

		Searched for a Basic Health Unit		PRc (95%CI)	PRa (95%CI)
		Yes % (95%CI)	No % (95%CI)		
Total		74.08 (73.21–74.94)	25.92 (25.06–26.79)		
Sex					
	Female	76.17 (75.12–77.2)	23.83 (22.8–24.88)	*	*
	Male	71.53 (70.34–72.69)	28.47 (27.31–29.66)	0.94 (0.92–0.96)	0.94 (0.92–0.96)
Age (years)					
	13–15	74.03 (72.98–75.06)	25.97 (24.94–27.02)	*	*
	16–17	74.17 (72.79–75.51)	25.83 (24.49–27.21)	1.00 (0.98–1.02)	0.99 (0.97–1.01)
Skin color					
	White	70.7 (69.4–71.97)	29.3 (28.03–30.6)	*	*
	Black	72.94 (70.48–75.27)	27.06 (24.73–29.52)	1.03 (1.00–1.07)	0.99 (0.96–1.03)
	Brown	77.27 (76.06–78.44)	22.73 (21.56–23.94)	1.09 (1.07–1.12)	1.06 (1.03–1.08)
	Others	75.0 (72.24–77.57)	25.0 (22.43–27.76)	1.06 (1.02–1.10)	1.03 (0.99–1.07)
Status					
	Private	58.5 (57.41–59.58)	41.5 (40.42–42.59)	*	*
	Public	77.04 (76.02–78.04)	22.96 (21.96–23.98)	1.32 (1.29–1.35)	1.32 (1.29–1.35)
Residence area					
	Urban	73.65 (72.73–74.54)	26.35 (25.46.27.27)	*	*
	Rural	79.86 (76.86–82.55)	20.14 (17.45–23.14)	1.08 (1.04–1.13)	1.05 (1.01–1.09)

* Reference group for calculating the prevalence ratio. %: prevalence; 95%CI: 95% confidence interval; PRc: crude prevalence ratio; PRa: prevalence ratio adjusted for sex, age and school status

Among the adolescents who sought a BHU, 88.61% (95%CI 88.05–89.14) were seen, however the proportion was lower among males (PRa: 0.98; 95%CI 0.97–0.99) and those with black skin color (PRa: 0.98; 95%CI 0.95–0.99) (Table 3).

**Table 3:** Assistance to students aged 13 to 17 years who sought the Basic Health Unit, according to sex, age, skin color, school status and area of residence. National School Health Survey, Brazil, 2019.

		Assisted at Basic Health Unit		PRc (95%CI)	PRa (95%CI)
		Yes % (95%CI)	No % (95%CI)		
Total		88.61 (88.05–89.14)	11.39 (10.86–11.95)		
Sex					
	Female	89. 31 (88.54–90.03)	10.69 (9.972–11.46)	*	*
	Male	87.73 (86.8–88.6)	12.27 (11.4–13.2)	0.98 (0.97–1.00)	0.98 (0.97–0.99)
Age (years)					
	13–15	88.59 (87.9–89.24)	11.41 (10.76–12.1)	*	*
	16–17	88.65 (87.71–89.52)	11.35 (10.48–12.29)	1.00 (0.99–1.01)	1.00 (0.99–1.01)
Skin color					
	White	89.04 (88.14–89.87)	10.96 (10.13–11.86)	*	*
	Black	86.89 (85.12–88.48)	13.11 (11.52–14.88)	0.98 (0.95–1.00)	0.98 (0.95–0.99)
	Brown	88.84 (87.98–89.65)	11.16 (10.35–12.02)	1.00 (0.98–1.01)	1.00 (0.98–1.01)
	Others	87.82 (85.64–89.71)	12.18 (10.29–14.36)	0.99 (0.98–1.01)	0.99 (0.96–1.01)
Status					
	Private	88.81 (88.11–89.47)	11.19 (10.53–11.89)	*	*
	Public	88.57 (87.92–89.19)	11.43 (10.81–12.08)	1.00 (0.99–1.01)	1.00 (0.99–1.01)
Residence area					
	Urban	88.65 (88.07–89.19)	11.35 (10.81–11.93)	*	*
	Rural	88.17 (85.65–90.3)	11.83 (9.70–14.35)	0.99 (0.97–1.02)	1.00 (0.97–1.02)

* Reference group for calculating the prevalence ratio; %: percentage; 95%CI: 95% confidence interval; PRc: crude prevalence ratio; PRa: prevalence ratio adjusted for sex, age and school status.

The main reasons why adolescents sought the BHU were: vaccination (27.93%; 95%CI 27.07–28.81); disease (19.98%; 95%CI 19.21–20.79); dental care (8.65%; 95%CI 8.15–9.17); accident or injury, physical therapy or rehabilitation (77.0%; 95%CI 6.56–7.46); support for weight control (6.29%; 95%CI 5.84–6.77); looking for a psychologist or mental health professional (3.36%; 95%CI 3.06–3.68); and other types of assistance, which included support to smoking cessation; contraceptive methods; HIV, syphilis or hepatitis B test; prenatal care/pregnancy test; request for a medical certificate (26.79%; 95%CI 25.93–27.66).

## DISCUSSION

This study identified that 56.56% of adolescent students sought a health service or professional to care for their own health. This demand was lower among males, with black, brown, yellow skin color and indigenous ethnicity, enrolled in public school and who resided in rural areas. The most frequently sought type of service was the BHU. Of the adolescents who sought BHUs, 88.61% were assisted and vaccination was the main reason for this demand.

More than half of the adolescents looked for some health service or professional, which shows a high demand in this age group. This highlights the importance of planning, reception and the quality of services provided to this public^11^. The priority actions of services for the comprehensive care of adolescents should include health education, immunization, nutrition and psychological support, considering their biological, emotional and social development, which is also fundamental for the success of the sustainable development agenda^12,13^.

This study showed that male adolescents sought health services less frequently than female ones. Sociocultural issues make it difficult to adhere to care and practices for health promotion and prevention of diseases and injuries^14,15^. Historically, men are seen as virile, invulnerable and strong, and demanding for health services can be associated with weakness, fear and insecurity^16^. Therefore, the importance of expanding and improving access to and quality of health services is reinforced to serve them in their plurality and in a resolute way, contributing to the acceptance and adherence to services and health promotion actions^17^. The importance of raising awareness about the demand for health services and professionals and the early encouragement of the development of healthy habits is added to this, as it will have repercussions in adult life^18^.

The search for and use of health services are also determined by situations of social vulnerability^19^. In this study, adolescents with black, brown, yellow skin color and indigenous ethnicity, enrolled in public schools and living in rural areas were the ones who least sought health services or professionals. This reflects the gaps in care due to socioeconomic and housing inequalities, as well as poor infrastructure, lack of transportation and difficulties for the State to cover all health demands in the most remote areas^20^. According to the 2019 National Health Survey (PNS), health care demand was lower among the low-income population and higher among those people with white skin and complete higher education^21^. A study with data from the National Household Sample Survey (PNAD) also showed that the use of health services is uneven and higher socioeconomic classes have better access^22^.

It is important to mention that students from private schools sought mainly doctors’ office or private clinics, which can be explained by better socioeconomic conditions, having a health insurance plan and greater access to private health services by the higher-income population^23^. Hence the importance of investing and prioritizing the Unified Health System (Sistema Único de Saúde — SUS) to expand the offer and improve the quality of health services and its infrastructure, ensuring the principles of universalization, equity and integrality.

The broad access to primary health services by adolescents is a finding similar to those of other studies^11,18^, which represents a step forward for the SUS, as it expands comprehensive health care and acts preventively. In this context, the BHUs, together with the Family Health Strategy teams, reorient the health care model and develop actions at the individual and collective levels, encompassing health promotion and protection, prevention of diseases and injuries, diagnosis, treatment, rehabilitation and health maintenance, being the population's first point of contact with the health system^24^. The BHU is also a primordial space for continuous, coordinated and oriented care for adolescents, which enables encounters and intersubjective exchanges that can produce dialogic relationships and promote care networks^25^. The improvement and expansion of the Primary Health Care network has a positive impact on the promotion of comprehensive health of Brazilian adolescents.

So, the present study showed that students from public schools seek BHUs more often and most of them are assisted, which shows the BHUs’ role in guaranteeing the health rights of different population groups^26^, in addition to fulfilling the principle of universality, in which everyone has the right to health actions and services. Despite the expansion of coverage and improvement of the quality of Primary Health Care services in Brazil, there are still challenges that require a continuous capacity for innovation in the formulation and implementation of health policies, models and practices^27^. Added to this, the underfunding of the SUS and the implementation of austerity policies such as the approval of Constitutional Amendment 95/2016, which reduced investments in social and health policies, contribute to the reduction in supply of services, the worsening of health indicators and the increase of inequalities^28^.

Changes are also needed to improve the care offered to adolescents, including training of professionals to deal with and meet their needs, and the inclusion of practices that advocate dialogue, considering real demands of this public and ensuring their rights. Available appointment dates on the service's schedule and qualified reception without prejudice are expected elements of a comprehensive, effective and ethical Primary Health Care^4^.

Among the various individual and collective actions carried out at the BHUs, vaccination is one of the leading services. The SUS, through the National Immunization Program (PNI), guarantees the vaccination of the entire Brazilian population with equity, effectiveness, efficiency and safety, in addition to offering, in the National Vaccination Calendar, all vaccines recommended by the World Health Organization and special immunobiologicals for groups at higher risk^29^. Vaccination actions in Brazil are an important tool to promote the principles of SUS, enabling the poorest municipalities in Brazil to comply with the same vaccination schedule as the richest ones^30^.

It is highlighted that schools are important for health promotion actions, for creating healthy environments and for consolidating public policies. As an example, there is the Health at School Program (PSE), created in 2007, consolidated as an important strategy for health promotion and disease prevention, having a positive impact on quality of life, learning conditions, and building citizenship. School health promotion initiatives are effective and can be enhanced by the active participation of Family Health teams^31^.

The development of actions that encourage the engagement of adolescents in continuous disease prevention and health promotion activities, with dynamic and proactive approaches, enables them to form autonomy and makes them co-responsible for their health^32^.

Some of the limitations of the study were memory bias and the self-administered questionnaire, which can lead to incorrect interpretations of questions by students and underestimations or overestimations of indicators. However, PeNSE was based on the main international surveys, such as the Global School-Based Student Health Survey, the Health Behavior in School-Aged Children and the Youth Risk Behavior Surveillance System, whose questionnaire was validated with satisfactory results in the reproducibility and validity analyses. Also, the research investigated students regularly enrolled and attending Brazilian education networks, excluding adolescents who do not have this educational bond and who may be more vulnerable. However, PeNSE also covers schools located in indigenous areas and places with remote access, with an increase in the sample of the 2019 edition, which made it possible to disaggregate it by large regions, FUs and municipalities within capitals. Therefore, even with limitations, the research represents the reality of adolescents aged 13 to 17 who attend school.

The results showed that adolescents search for and use health services, but that health inequalities still persist, mainly due to socioeconomic issues. This reinforces the need for investment in public policies, in Primary Health Care and in the SUS, in addition to expanding the offer and improving the quality of services, especially among adolescents, as they are in a phase of important psychobiological and social transformations, which makes them a strategic group for health promotion and prevention of diseases and injuries. Thus, adolescent care should encompass counseling, health promotion and disease prevention actions, considering their particularities, vulnerabilities, as well as the familial, social, economic and cultural context they are in.
