# Supplementary material for: Demand and use of health services by Brazilian adolescents, according to the National School Health Survey 2019
Source: Rev Bras Epidemiol. 2023 Apr 21;26(Suppl 1):e230008. doi: 10.1590/1980-549720230008.supl.1 (PMC10176729; doi:10.1590/1980-549720230008.supl.1)
Supplement: Translation

# Translation: Procura e utilização dos serviços de saúde por adolescentes brasileiros, segundo a Pesquisa Nacional de Saúde do Escolar de 2019

**DOI:** 10.1590/1980-549720230008.supl.1.1

**Authors:** Alanna Gomes da Silva, Crizian Saar Gomes, Alan Cristian Marinho Ferreira, Deborah Carvalho Malta

**Affiliations:** I Universidade Federal de Minas Gerais, Escola de Enfermagem, Programa de Pós-Graduação em Enfermagem – Belo Horizonte (MG), Brasil.; II Universidade Federal de Minas Gerais, Faculdade de Medicina, Programa de Pós-Graduação em Saúde Pública – Belo Horizonte (MG), Brasil.

**Keywords:** Saúde do adolescente, Acesso aos serviços de saúde, Sistema único de saúde, Promoção da saúde

## Abstract

**Objetivo:**

Analisar a procura e a utilização dos serviços de saúde por adolescentes brasileiros, segundo características sociodemográficas.

**Métodos:**

Estudo transversal com dados da Pesquisa Nacional de Saúde do Escolar de 2019. A amostra foi composta de 124.898 adolescentes de 13 a 17 anos. Foram calculadas as razões de prevalência bruta e ajustada (RPaj) por sexo, idade e dependência administrativa e seus intervalos de confiança de 95% (IC95%) das variáveis “procura por algum serviço ou profissional de saúde”, “procura por alguma Unidade Básica de Saúde” e “atendimento na Unidade Básica de Saúde”, utilizando a regressão de Poisson com variância robusta.

**Resultados:**

A procura por algum serviço de saúde foi relatada por 56,56% (IC95% 55,82–57,29) dos adolescentes, sendo menor entre o sexo masculino (RPaj: 0,95; IC95% 0,94–0,95); aqueles com cor da pele preta (RPaj: 0,95; IC95% 0,94–0,97), parda (RPaj: 0,97; IC95% 0,96–0,98), amarela e indígena (RPaj: 0,95; IC95% 0,94–0,97); estudantes de escolas públicas (RPaj: 0,90; IC95% 0,89–0,90) e residentes da zona rural (RPaj: 0,96; IC95% 0,94–0,98). A Unidade Básica de Saúde foi procurada por 74,08% (IC95% 73,21–74,94) dos adolescentes e foi mais frequente entre aqueles de cor da pele parda (RPaj: 1,06; IC95% 1,03–1,08), de escolas públicas (RPaj: 1,32; IC95% 1,29–1,35) e residentes da zona rural (RPaj: 1,05; IC95%1,01–1,09). O principal motivo da procura pela Unidade Básica de Saúde foi a vacinação (27,93%; IC95% 27,07–28,81).

**Conclusão:**

Mais da metade dos adolescentes procurou algum serviço de saúde, mostrando elevada demanda dessa população. Contudo, ainda persistem as desigualdades, o que nos alerta sobre a importância do planejamento, do acolhimento e da qualidade da atenção prestada aos adolescentes.

## INTRODUÇÃO

Durante o período da adolescência, os indivíduos passam por um processo dinâmico e complexo de maturação e transformações física, hormonal, psíquica e social, que os podem predispor a novas experiências e comportamentos de risco para a saúde^1,2^.

A cada ano, milhares de adolescentes morrem por causas evitáveis^3^, o que reforça a necessidade de os serviços fornecerem atenção integral à sua saúde e o acesso às ações individuais e coletivas de promoção da saúde, prevenção de doenças e agravos^4,5^. Para que os adolescentes sejam atendidos de forma integral, é necessária uma rede organizada, planejada e que tenha a participação dos atores sociais e de profissionais de saúde capacitados para atendê-los, considerando, sobretudo, os contextos socioeconômicos e culturais^6^.

A procura por serviço de saúde pode ser definida como a entrada do indivíduo nesse sistema, enquanto a utilização compreende a interação entre profissionais e usuários, que pode se dar por contato direto, como as consultas e as hospitalizações, ou indireto, como a realização de exames^7^.

A procura e a utilização dos serviços de saúde podem ser influenciadas pelos determinantes sociais, visto que os adolescentes de baixa renda, socialmente desfavorecidos ou marginalizados procuram menos os serviços de saúde. Em contrapartida, aqueles com cor da pele branca, estudantes de escola privada e com escolaridade materna elevada procuram mais os serviços e os profissionais de saúde^8,9^. Soma-se a isso a dificuldade dos profissionais em prestar um cuidado integral aos adolescentes, o que prejudica o acolhimento, o estabelecimento do vínculo e a qualidade do atendimento ofertado^4^.

Estudos que utilizaram os dados da Pesquisa Nacional de Saúde do Escolar (PeNSE) mostraram que 48,0% (IC95% 47,6–48,5) dos adolescentes procuraram por algum serviço ou profissional de saúde em 2012^8^ e 56,7% (IC95% 55,2–58,3), em 2015^9^, o que revela aumento ao longo dos anos. Contudo, o monitoramento da procura e utilização dos serviços de saúde deve ser contínuo para possibilitar a tomada de decisões, fortalecer a gestão e as políticas públicas e reconhecer as demandas dessa população, possibilitando a identificação dos fatores que influenciam a relação com os serviços e os profissionais de saúde. Assim, espera-se que um cuidado contínuo, coordenado e orientado aos adolescentes seja prestado e aprimorado, para a promoção dos seus direitos e a redução das desigualdades em saúde.

Nesse sentido, este estudo teve como objetivo analisar a procura e a utilização dos serviços de saúde por adolescentes brasileiros, segundo características sociodemográficas.

## MÉTODOS

### Delineamento do estudo

Trata-se de um estudo transversal que utilizou os dados da PeNSE realizada em 2019.

### Cenário

A PeNSE é um inquérito realizado com escolares adolescentes, conduzido pelo Instituto Brasileiro de Geografia e Estatística (IBGE) em parceria com o Ministério da Saúde e o apoio do Ministério da Educação. A pesquisa compõe o Sistema de Vigilância dos Fatores de Risco e Proteção das Doenças Crônicas Não Transmissíveis (DCNT) do Brasil e foi o primeiro inquérito nacional que abordou diversos aspectos da vida dos adolescentes, como hábitos, cuidados, fatores de risco e proteção para a sua saúde^10^.

O plano amostral da pesquisa foi por conglomerados em dois estágios, cujas escolas correspondem ao primeiro estágio de seleção e as turmas de alunos matriculados ao segundo. O conjunto dos estudantes das turmas selecionadas formaram a amostra de alunos. A amostra foi dimensionada para estimar parâmetros populacionais para os estudantes de 13 a 17 anos matriculados e que frequentavam regularmente as escolas públicas e privadas, para os seguintes níveis geográficos: Brasil, grandes regiões, unidades da Federação (UF), municípios das capitais e Distrito Federal. Detalhes sobre o processo de amostragem são fornecidos na publicação da PeNSE^10^.

Em 2019, foram coletados os dados de 4.242 escolas, 6.612 turmas, com 189.857 alunos matriculados e 183.264 alunos frequentes, sendo 159.245 questionários válidos e 125.123 questionários analisados^10^.

A base de dados e os questionários da PeNSE estão disponíveis para acesso e uso público no *site* do IBGE: https://www.ibge.gov.br/estatisticas/sociais/saude/9134-pesquisa-nacional-de-saude-do-escolar.html?edicao=31442&t=resultados.

### Participantes

Participaram da pesquisa escolares adolescentes de 13 a 17 anos de idade, matriculados e com frequência regular do 7^o^ ao 9^o^ ano do ensino fundamental e da 1ᵃ à 3ᵃ série do ensino médio, incluindo os cursos técnicos com ensino médio integrado e os cursos normal/magistério, de todos os turnos, de escolas públicas e privadas do Brasil^10^.

### Coleta dos dados

A coleta dos dados ocorreu de abril a setembro de 2019 e foi realizada com o dispositivo móvel de coleta, que corresponde a um *smartphone* com os questionários estruturados da pesquisa. O técnico do IBGE distribuiu os aparelhos aos alunos presentes no dia das entrevistas e orientou-os quanto ao seu manuseio. O questionário do aluno foi autoaplicável e possuía orientações específicas de preenchimento^10^.

### Variáveis do estudo

Este estudo incluiu variáveis referentes à procura e utilização dos serviços de saúde e variáveis sociodemográficas que estão descritas a seguir.

Procura por algum serviço ou profissional de saúde: prevalência de adolescentes que procuraram por algum serviço ou profissional de saúde para atendimento relacionado à saúde. “Nos últimos 12 meses você procurou algum serviço ou profissional de saúde para atendimento relacionado à própria saúde?” Opções de resposta: sim; não.

Serviço procurado com mais frequência: proporção do tipo de serviço de saúde procurado com mais frequência pelos adolescentes para atendimento relacionado à própria saúde. “Nos últimos 12 meses, qual foi o serviço de saúde que você procurou com mais frequência?” Opções de resposta: Unidade Básica de Saúde (UBS); consultório médico ou clínica particular; hospital; outros (consultório odontológico; consultório de outro profissional de saúde; serviços de especialidades médicas ou policlínica; pronto-socorro, emergência ou Unidade de Pronto Atendimento (UPA); laboratório ou clínica para exames complementares; serviço de atendimento domiciliar; farmácia).

Procura por alguma UBS: proporção de adolescentes que procurou alguma UBS para atendimento relacionado à própria saúde nos últimos 12 meses. “Nos últimos 12 meses, você procurou por alguma UBS (Centro ou Posto de Saúde ou Unidade de Saúde da Família/Programa Saúde da Família — PSF)?” Opções de resposta: sim; não.

*Atendimento na UBS:* proporção de adolescentes que recebeu atendimento quando procurou alguma UBS. “Você foi atendido na última vez que procurou alguma UBS (Centro ou Posto de Saúde ou Unidade de Saúde da Família — PSF)?” Opções de resposta: sim; não.

Motivo da procura pela UBS: proporção do principal motivo de os adolescentes terem procurado alguma UBS. “Qual foi o principal motivo da sua procura na UBS (Centro ou Posto de Saúde ou Unidade de Saúde da Família — PSF) nesta última vez?” Opções de resposta: apoio para controle de peso (ganhar ou perder); acidente ou lesão, reabilitação ou fisioterapia; dentista ou outro profissional de saúde bucal; psicólogo ou outro profissional de saúde mental; vacinação; doença; outros (apoio para parar de fumar, obter métodos contraceptivos, teste HIV, sífilis ou hepatite B, pré-natal/teste para gravidez, solicitação de atestado médico).

A Figura 1 apresenta o fluxo do questionário da PeNSE quanto aos indicadores supracitados.

**Figura 1:** Fluxograma das perguntas relacionadas à procura e utilização dos serviços de saúde pelos escolares de 13 a 17 anos. PeNSE, Brasil, 2019.

Variáveis sociodemográficas: sexo (masculino e feminino); faixa etária (de 13 a 15; 16 e 17 anos); cor da pele (branca, preta, parda e outros — amarela e indígena); dependência administrativa da escola (privada e pública) e local de moradia (urbano e rural).

### Análises dos dados

Foram estimadas as prevalências e os respectivos intervalos de confiança de 95% (IC95%). Para as variáveis “procura por algum serviço ou profissional de saúde”, “procura por alguma UBS” e “atendimento na UBS” foram calculadas as proporções segundo as variáveis sociodemográficas e as razões de prevalência (RP) bruta (RPb) e ajustada (RPaj) por sexo, idade e dependência administrativa da escola^8,9^ e os seus IC95%, utilizando modelos de regressão de Poisson com variância robusta. Consideraram-se significativos os resultados com o valor p menor ou igual a 0,05. Para todas as análises, consideraram-se os pesos de pós-estratificação.

As análises foram realizadas no Data Analysis and Statistical Software (Stata), versão 14.2, empregando o módulo *survey*, que considera os pesos de pós-estratificação.

### Aspectos éticos

Participaram da pesquisa os alunos que concordaram com o Termo de Consentimento Livre e Esclarecido (TCLE), visualizado na primeira página do questionário no dispositivo móvel de coleta.

A PeNSE foi aprovada pela Comissão Nacional de Ética em Pesquisa para Seres Humanos do Ministério da Saúde (Parecer n^o^ 3.249.268, de 8 de abril de 2019).

## RESULTADOS

Foram avaliados 124.898 escolares adolescentes com idade entre 13 e 17 anos. Deles, a maioria era do sexo feminino (50,7%; IC95% 49,9–51,4); com idade entre 13 e 15 anos (64,7%; IC95% 63,2–66,01); com cor da pele parda (43,6%; IC95% 42,8–44,3), seguida de branca (36,0%; IC95% 35,1–36,8), preta (13,6%; IC95% 13,0–14,1), amarela e indígena (6,9%; IC95% 6,5–7,2); residente da zona urbana (92,4%; IC95% 90,6–93,6); e estudante de escolas públicas (85,5%; IC95% 85,1–85,9).

A procura por algum serviço ou profissional de saúde para atendimento relacionado à própria saúde foi relatada por 56,56% (IC95% 55,82–57,29) dos adolescentes e foi menor entre o sexo masculino (RPaj: 0,95; IC95% 0,94–0,95); aqueles com cor da pele preta (RPaj: 0,95; IC95% 0,94–0,97), parda (RPaj: 0,97; IC95% 0,96–0,98), amarela e indígena (RPaj: 0,95; IC95% 0,94–0,97); estudantes de escolas públicas (RPaj: 0,90; IC95% 0,89–0,90); e residentes da zona rural (RPaj: 0,96; IC95% 0,94–0,98). Por outro lado, a procura foi maior entre os adolescentes com 16 e 17 anos de idade (RPaj: 1,03; IC95% 1,02–1,04) (Tabela 1).

**Tabela 1:** Procura por algum serviço ou profissional de saúde para atendimento relacionado à própria saúde, segundo sexo, idade, raça, dependência administrativa da escola e local de moradia. Pesquisa Nacional de Saúde do Escolar, Brasil, 2019.

		Procura por serviço ou profissional de saúde		RPb (IC95%)	RPaj (IC95%)
		Sim % (IC95%)	Não % (IC95%)		
Total		56,56 (55,82–57,29)	43,44 (42,71–44,18)		
Sexo					
	Feminino	60,72 (59,85–61,58)	39,28 (38,42–40,15)	*	*
	Masculino	52,23 (51,27–53,19)	47,77 (46,81–48,73)	0,95 (0,94–0,95)	0,95 (0,94–0,95)
Idade (anos)					
	13 a 15	55,12 (54,08–56,14)	44,88 (43,86–45,92)	*	*
	16 e 17	59,20 (58,28–60,12)	40,8 (39,88–41,72)	1,03 (1,02–1,04)	1,03 (1,02–1,04)
Raça					
	Branca	61,25 (60,16–62,34)	38,75(37,66–39,84)	*	*
	Preta	51,45 (49,64–53,25)	48,55 (46,75–50,36)	0,94 (0,93–0,95)	0,95 (0,94–0,97)
	Parda	55,03 (54,03–56,02)	44,97 (43,98–45,97)	0,96 (0,95–0,97)	0,97 (0,96–0,98)
	Outros	51,93 (49,57–54,27)	48,07 (45,73–50,43)	0,94 (0,93–0,96)	0,95 (0,94–0,97)
Dependência administrativa					
	Privada	71,55(70,73–72,36)	28,45 (27,64–29,27)	*	*
	Pública	54,02 (53,16,54,87)	45,98 (45,13–46,84)	0,90 (0,89–0,90)	0,90 (0,89–0,90)
Local de moradia					
	Urbano	57,27 (56,50–58,04)	42,73 (41,96–43,5)	*	*
	Rural	47,9 (45,13–50,68)	52,1 (49,32–54,87)	0,94 (0,92–0,96)	0,96 (0,94–0,98)

* Grupo de referência para o cálculo da razão de prevalência. %: prevalência; IC95%: intervalo de 95% de confiança; RPb: razão de prevalência bruta; RPaj: razão de prevalência ajustada por sexo, idade e dependência administrativa da escola.

Acerca do tipo de serviço de saúde procurado com mais frequência pelos adolescentes, os resultados demonstraram que 35,97% (IC95% 34,98–36,97) procuraram pela UBS. Destaca-se que os alunos de escola pública procuraram com maior frequência a UBS (40,75%; IC95% 39,56–41,95) e os de escola privada, o consultório médico ou clínica particular (38,49%; IC95% 37,52–39,46) (Figura 2).

**Figura 2:** Tipo de serviço procurado com mais frequência pelos escolares de 13 a 17 anos para atendimento relacionado à própria saúde, segundo dependência administrativa. Pesquisa Nacional de Saúde do Escolar, Brasil, 2019.

Quando questionados especificamente sobre a procura por alguma UBS, 74,08% (IC95% 73,21–74,94) dos adolescentes procuraram por atendimento nesse serviço, principalmente os de cor da pele parda (RPaj: 1,06; IC95% 1,03–1,08), de escolas públicas (RPaj: 1,32; IC95% 1,29–1,35) e residentes da zona rural (RPaj: 1,05; IC95%1,01–1,09). Em contrapartida, houve menos procura pelo sexo masculino (RPaj: 0,94; IC95% 0,92–0,96). Não houve diferença segundo faixa etária (Tabela 2).

**Tabela 2:** Escolares de 13 a 17 anos que procuram por alguma Unidade Básica de Saúde, segundo sexo, idade, raça, dependência administrativa da escola e local de moradia. Pesquisa Nacional de Saúde do Escolar, Brasil, 2019.

		Procura por alguma Unidade Básica de Saúde		RPb (IC95%)	RPaj (IC95%)
		Sim % (IC95%)	Não % (IC95%)		
Total		74,08 (73,21–74,94)	25,92 (25,06–26,79)		
Sexo					
	Feminino	76,17 (75,12–77,2)	23,83 (22,8–24,88)	*	*
	Masculino	71,53 (70,34–72,69)	28,47 (27,31–29,66)	0,94 (0,92–0,96)	0,94 (0,92–0,96)
Idade (anos)					
	13 a 15	74,03 (72,98–75,06)	25,97 (24,94–27,02)	*	*
	16 e 17	74,17 (72,79–75,51)	25,83 (24,49–27,21)	1,00 (0,98–1,02)	0,99 (0,97–1,01)
Raça					
	Branca	70,7 (69,4–71,97)	29,3 (28,03–30,6)	*	*
	Preta	72,94 (70,48–75,27)	27,06 (24,73–29,52)	1,03 (1,00–1,07)	0,99 (0,96–1,03)
	Parda	77,27 (76,06–78,44)	22,73 (21,56–23,94)	1,09 (1,07–1,12)	1,06 (1,03–1,08)
	Outros	75,0 (72,24–77,57)	25,0 (22,43–27,76)	1,06 (1,02–1,10)	1,03 (0,99–1,07)
Dependência administrativa					
	Privada	58,5 (57,41–59,58)	41,5 (40,42–42,59)	*	*
	Pública	77,04 (76,02–78,04)	22,96 (21,96–23,98)	1,32 (1,29–1,35)	1,32 (1,29–1,35)
Local de moradia					
	Urbano	73,65 (72,73–74,54)	26,35 (25,46,27,27)	*	*
	Rural	79,86 (76,86–82,55)	20,14 (17,45–23,14)	1,08 (1,04–1,13)	1,05 (1,01–1,09)

* Grupo de referência para o cálculo da razão de prevalência. %: porcentagem; IC95%: intervalo de 95% de confiança; RPb: razão de prevalência bruta; RPaj: razão de prevalência ajustada por sexo, idade e dependência administrativa da escola.

Entre os adolescentes que procuraram uma UBS, 88,61% (IC95% 88,05–89,14) deles foram atendidos, contudo a procura foi menor entre o sexo masculino (RPaj: 0,98; IC95% 0,97–0,99) e aqueles com a cor da pele preta (RPaj: 0,98; IC95% 0,95–0,99) (Tabela 3).

**Tabela 3:** Atendimento dos escolares de 13 a 17 anos que procuraram a Unidade Básica de Saúde, segundo sexo, idade, raça, dependência administrativa da escola e local de moradia. Pesquisa Nacional de Saúde do Escolar, Brasil, 2019.

		Atendidos na UBS		RPb (IC95%)	RPaj (IC95%)
		Sim % (IC95%)	Não % (IC95%)		
Total		88,61 (88,05–89,14)	11,39 (10,86–11,95)		
Sexo					
	Feminino	89,31 (88,54–90,03)	10,69 (9,97–11,46)	*	*
	Masculino	87,73 (86,8–88,6)	12,27 (11,4–13,2)	0,98 (0,97–1,00)	0,98 (0,97–0,99)
Idade (anos)					
	13 a 15	88,59 (87,9–89,24)	11,41 (10,76–12,1)	*	*
	16 e 17	88,65 (87,71–89,52)	11,35 (10,48–12,29)	1,00 (0,99–1,01)	1,00 (0,99–1,01)
Raça					
	Branca	89,04 (88,14–89,87)	10,96 (10,13–11,86)	*	*
	Preta	86,89 (85,12–88,48)	13,11 (11,52–14,88)	0,98 (0,95–1,00)	0,98 (0,95–0,99)
	Parda	88,84 (87,98–89,65)	11,16 (10,35–12,02)	1,00 (0,98–1,01)	1,00 (0,98–1,01)
	Outros	87,82 (85,64–89,71)	12,18 (10,29–14,36)	0,99 (0,98–1,01)	0,99 (0,96–1,01)
Dependência administrativa					
	Privada	88,81 (88,11–89,47)	11,19 (10,53–11,89)	*	*
	Pública	88,57 (87,92–89,19)	11,43 (10,81–12,08)	1,00 (0,99–1,01)	1,00 (0,99–1,01)
Local de moradia					
	Urbano	88,65 (88,07–89,19)	11,35 (10,81–11,93)	*	*
	Rural	88,17 (85,65–90,3)	11,83 (9,70–14,35)	0,99 (0,97–1,02)	1,00 (0,97–1,02)

* Grupo de referência para o cálculo da razão de prevalência. UBS: Unidade Básica de Saúde; %: porcentagem; IC95%: intervalo de 95% de confiança; RPb: razão de prevalência bruta; RPaj: razão de prevalência ajustada por sexo, idade e dependência administrativa da escola.

Os principais motivos de os adolescentes terem procurado a UBS foram: vacinação (27,93%; IC95% 27,07–28,81); doença (19,98%; IC95% 19,21–20,79); atendimento odontológico (8,65%; IC95% 8,15–9,17); acidente ou lesão, fisioterapia ou reabilitação (7,0%; IC95% 6,56–7,46); apoio para o controle de peso (6,29%; IC95% 5,84–6,77); procura por psicólogo ou profissional de saúde mental (3,36%; IC95% 3,06–3,68); e outros atendimentos, que incluíram apoio para parar de fumar, obter métodos contraceptivos, teste HIV, sífilis ou hepatite B, pré-natal/teste para gravidez ou solicitação de atestado médico (26,79%; IC95% 25,93–27,66).

## DISCUSSÃO

Este estudo verificou que 56,56% dos escolares adolescentes procuraram algum serviço ou profissional de saúde para atendimento relacionado à própria saúde. Essa procura foi menor entre o sexo masculino, aqueles com cor da pele preta, parda, amarela e indígena, estudantes de escolas públicas e residentes da zona rural. O tipo de serviço procurado com mais frequência foi a UBS. Dos adolescentes que a procuram, 88,61% foram atendidos e a vacinação foi o principal motivo dessa procura.

Mais da metade dos adolescentes procurou algum serviço ou profissional de saúde, o que revela a elevada demanda dessa faixa etária. Isso alerta sobre a importância do planejamento, do acolhimento e da qualidade dos serviços prestados a esse público^11^. As ações prioritárias dos serviços para o atendimento integral dos adolescentes devem incluir educação em saúde, imunização, nutrição e apoio psicológico, considerando o seu desenvolvimento biológico, emocional e social, que são também fundamentais para o sucesso da agenda de desenvolvimento sustentável^12,13^.

Este estudo demonstrou que os adolescentes do sexo masculino procuraram menos os serviços de saúde em comparação com os do sexo feminino. As questões socioculturais dificultam a adesão aos cuidados e às práticas de promoção da saúde e prevenção de doenças e agravos^14,15^. Historicamente, o homem é visto como viril, invulnerável e forte, e a procura pelo serviço de saúde pode associá-lo a fraqueza, medo e insegurança^16^. Assim, reforça-se a importância de ampliar e melhorar o acesso e a qualidade dos serviços de saúde para atendê-los na sua pluralidade e de forma resolutiva, contribuindo para o acolhimento e a adesão aos serviços e às ações de promoções da saúde^17^. Somam-se a isso a importância da conscientização para a procura dos serviços e profissionais de saúde e o estímulo precoce ao desenvolvimento de hábitos saudáveis, os quais terão reflexos na vida adulta^18^.

A procura e a utilização dos serviços de saúde também são determinadas por situações de vulnerabilidade social da população^19^. Neste estudo, os adolescentes com cor da pele preta, parda, amarela e indígena, estudantes de escolas públicas e moradores da zona rural foram os que menos procuraram os serviços ou profissionais de saúde. Isso reflete os vazios assistenciais devidos às desigualdades socioeconômicas e locais de moradia, em função de menor infraestrutura, escassez de transporte e dificuldades do Estado para cobrir todas as demandas de saúde nas áreas mais remotas^20^. De acordo com a Pesquisa Nacional de Saúde (PNS) de 2019, a proporção da procura de atendimento de saúde foi menor entre a população de baixa renda e maior entre aqueles com a cor da pele branca e com ensino superior completo^21^. Um estudo com dados da Pesquisa Nacional por Amostra de Domicílios (PNAD) também evidenciou que o uso dos serviços de saúde é desigual e favorece as classes socioeconômicas mais elevadas^22^.

Torna-se importante mencionar que os estudantes de escolas privadas procuraram principalmente o consultório médico ou clínica particular, o que pode ser justificado pelas melhores condições socioeconômicas, pela posse de plano de saúde e pelo maior acesso a serviços particulares de saúde entre a população de maior renda^23^. Por isso, a importância do investimento e da priorização no Sistema Único de Saúde (SUS) da ampliação e melhoria da oferta, da qualidade dos serviços de saúde e da sua infraestrutura, para que se façam cumprir os princípios da universalização, da equidade e da integralidade.

O amplo acesso aos serviços primários de saúde pelos adolescentes é um achado semelhante aos de outros estudos^11,18^, o que representa um avanço do SUS, por ampliar o cuidado integral à saúde e atuar preventivamente. Nesse contexto, as UBS com as equipes de Saúde da Família reorientam o modelo assistencial de saúde e desenvolvem ações no âmbito individual e coletivo, que abrangem a promoção e a proteção da saúde, a prevenção de doenças e agravos, o diagnóstico, o tratamento, a reabilitação e a manutenção da saúde, sendo o primeiro ponto de contato da população com o sistema de saúde^24^. A UBS também se constitui como espaço primordial para o cuidado contínuo, coordenado e orientado aos adolescentes, que possibilita encontros e trocas intersubjetivas capazes de produzir relações dialógicas e promover redes de cuidado^25^. O aprimoramento e a expansão da rede de Atenção Primária à Saúde (APS) impacta positivamente a promoção integral à saúde dos adolescentes brasileiros.

Nessa direção, o presente estudo mostrou que os alunos de escolas públicas procuram mais as UBS e que a maior parte dos adolescentes que buscaram essas unidades receberam atendimento, o que mostra o seu papel na garantia dos direitos à saúde dos diferentes grupos populacionais^26^, além de cumprir o princípio da universalidade, segundo o qual todos têm direito às ações e serviços de saúde. Apesar da expansão da cobertura e da ampliação da qualidade de serviços da APS no Brasil, ainda emergem desafios, que exigem uma capacidade contínua de inovação na formulação e na implantação de políticas, modelos e práticas em saúde^27^. Somam-se a isso o subfinancimento do SUS e a implementação das políticas de austeridade como a aprovação da Emenda Constitucional nº 95/2016, que reduziu os investimentos em políticas sociais e de saúde; isso contribui para a redução da oferta dos serviços, a piora dos indicadores de saúde e o aumento das desigualdades^28^.

Mudanças também são necessárias para a melhoria do cuidado ofertado aos adolescentes, como a capacitação dos profissionais para lidar e atender as suas necessidades e a inclusão de práticas que preconizem o diálogo, que considerem as reais demandas desse público e garantam os seus direitos. O espaço na agenda dos serviços e o acolhimento qualificado e sem preconceitos são os elementos esperados de uma APS integral, efetiva e ética^4^.

Entre as diversas ações individuais e coletivas realizadas na UBS, a vacinação é um dos principais serviços oferecidos. O SUS, por meio do Programa Nacional de Imunizações (PNI), garante a vacinação de toda a população brasileira com equidade, efetividade, eficiência e segurança, além de oferecer, no Calendário Nacional de Vacinação, todas as vacinas recomendadas pela Organização Mundial da Saúde e os imunobiológicos especiais para grupos em situação de maior risco^29^. As ações de vacinação no Brasil constituem instrumento importante para a promoção dos princípios do SUS, possibilitando que os municípios mais pobres do Brasil cumpram o mesmo calendário vacinal que os mais ricos^30^.

Destaca-se que as escolas são importantes para a concretização de ações de promoção da saúde, para a criação de ambientes saudáveis e para a consolidação de políticas públicas. Como exemplo, tem-se o Programa Saúde na Escola (PSE), criado em 2007, consolidado como importante estratégia para a promoção da saúde e a prevenção de agravos, que tem impacto positivo na qualidade de vida, nas condições de aprendizado e na construção da cidadania. As iniciativas de promoção da saúde escolar são efetivas e podem ser potencializadas pela participação ativa das equipes de Saúde da Família^31^.

O desenvolvimento de ações que propiciem o engajamento dos adolescentes nas atividades contínuas de prevenção de doenças e de promoção à saúde, com abordagens dinâmicas e proativas, possibilita a formação da sua autonomia e torna-os corresponsáveis pela sua saúde^32^.

Entre as limitações do estudo, consideram-se o viés de memória e o questionário autoaplicado, que pode gerar interpretações incorretas das questões pelos escolares e subestimativas ou superestimativas dos indicadores estudados. Contudo, a PeNSE baseou-se nos principais inquéritos internacionais, como o Global School-Based Student Health Survey, o Health Behaviour in School-Aged Children e o Youth Risk Behavior Surveillance System, os quais apresentam validação do questionário, com resultados satisfatórios nas análises de reprodutibilidade e validade. Tem-se também o fato de a pesquisa investigar estudantes regularmente matriculados e frequentes nas redes de ensino do Brasil, excluindo os adolescentes que não possuem esse vínculo educacional e que podem apresentar maior vulnerabilidade. No entanto, a PeNSE abrange também as escolas localizadas em áreas indígenas e em locais de acesso remoto, havendo ampliação da amostra da edição de 2019, a qual possibilitou a sua desagregação por grandes regiões, UF e municípios das capitais. Assim, mesmo com as limitações, a pesquisa representa a realidade dos adolescentes de 13 a 17 anos que frequentam a escola.

Os resultados mostraram que os adolescentes procuram e utilizam os serviços de saúde, mas que ainda persistem as desigualdades nesse campo, principalmente pelas questões socioeconômicas. Isso reforça a necessidade de investimento em políticas públicas, na APS e no SUS, além de aumentar a oferta e a qualidade dos serviços, especialmente entre os adolescentes, que estão em uma fase de importantes transformações psicobiológicas e sociais, o que os torna um grupo estratégico para a promoção da saúde e a prevenção de doenças e agravos. Diante disso, a atenção ao adolescente deve proporcionar aconselhamento, ações de promoção à saúde e prevenção de agravos, considerando as particularidades, as vulnerabilidades e o contexto familiar, social, econômico e cultural em que eles estão inseridos.
